# Role of Heterojunction Engineering in Sputtered WO_3_/CuWO_4_ and WO_3_/CuWO_4_/TiO_2_ Thin Films for Enhanced Photocatalysis

**DOI:** 10.1021/acsomega.6c00156

**Published:** 2026-04-29

**Authors:** Lucas Caniati Escaliante, Nilton Francelosi Azevedo Neto, Luiz Felipe Kaezmarek Pedrini, Kleper de Oliveira Rocha, Jose Humberto Dias da Silva

**Affiliations:** † School of Sciences, Graduate Program in Materials Science and Technology − POSMAT, Universidade Estadual Paulista − UNESP, Bauru, São Paulo 17033-360, Brazil; ‡ Plasma and Processes Laboratory, Instituto de Tecnologia Aeronáutica − ITA, São José dos Campos, São Paulo 12228-900, Brazil; § School of Sciences, Chemistry Department, Universidade Estadual Paulista − UNESP, Bauru, São Paulo 17033-360, Brazil

## Abstract

Heterojunction engineering is a promising approach to overcoming the intrinsic limitations of individual semiconductor photocatalysts. In this context, WO_3_/CuWO_4_ and WO_3_/CuWO_4_/TiO_2_ heterojunctions were deposited by reactive magnetron sputtering. The resulting heterojunctions were subjected to annealing in air at 600 °C and systematically investigated, with respect to their structural, optical, and photocatalytic properties. The characterization of the developed samples, including X-ray diffraction, Rietveld refinement, Raman spectroscopy, scanning electron and atomic force microscopies (SEM and AFM), and optical spectroscopy, revealed the possibility of controlling the WO_3_/CuWO_4_ ratio, directly influencing the heterojunction band gap and surface morphology. Photocatalytic degradation of methylene blue under 405 nm LED illumination demonstrated that intermediate WO_3_/CuWO_4_ compositions achieved the highest efficiencies, which may be attributed to enhanced charge separation at the type-II heterojunction interface. Besides, the introduction of an ultrathin TiO_2_ overlayer (∼2 nm) further improved the dye degradation activity. This effect is attributed to defect passivation and selective hole transport. In contrast, with thicker TiO_2_ overlayers, the heterojunction contribution is suppressed, and the activity behavior is similar to that observed for bulk TiO_2_. These results highlight the critical role of precise stoichiometric control and surface overlayer engineering in optimizing the performance of oxide-based heterostructures, establishing sputtering deposition as a scalable strategy for photocatalytic applications in environmental remediation and solar-driven energy conversion.

## Graphical abstract

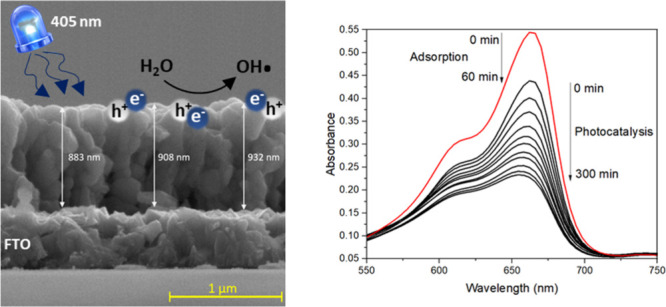

## Introduction

The increasing demand for sustainable energy and environmental remediation has positioned photocatalysis as one of the most promising technologies.^1^ This process enables the direct conversion of solar energy into specific chemical reactions,^2^ offering a green pathway for applications such as hydrogen generation through water splitting,^3^ CO_2_ reduction,^4^ and degradation of emerging pollutants.^5^

Among semiconductor photocatalysts, TiO_2_ has long been considered a benchmark material due to its chemical stability, low cost, and superior activity as compared to other photocatalysts.^6,7^ However, its wide band gap (∼3.2 eV) limits absorption in the UV region, which represents less than 5% of the solar spectrum.^8^ Accordingly, alternative strategies must be considered to harvest visible light, such as employing other semiconductors, such as WO_3_ and CuWO_4_.^9–11^ These materials extend the photoresponse into the visible range while maintaining adequate stability under operating conditions.^8,12–14^

CuWO_4_ has a triclinic structure.^15,16^ Its conduction band is composed of unoccupied 5d and 3d orbitals of W and Cu,^15^ respectively, and the valence band is mainly composed of hybridized states of O 2p^6^ and Cu 3d^9^.^17^ These valence band states cause a decrease in the bandgap energy relative to WO_3_, expanding the range of absorbed visible light.^15,17,18^ It is a potential alternative due to the smaller band gap (2.2–2.4 eV),^17,19^ absorbing visible light.^14,20^ Recently, it was reported that the photocatalytic performance of CuWO_4_ can be improved by coupling it with WO_3_, resulting in a type-II heterojunction. This configuration enhances charge carrier separation, suppresses recombination, and improves carrier mobility, mitigating limitations associated with intraband-gap Cu^2+/+^ surface states.^12,13,19,21–23^ While thin WO_3_/CuWO_4_ films were already deposited by spin coating,^24^ electrodeposition,^25^ and further improved via introduction of cocatalysts,^13^ plasmonic nanoparticles,^22^ or optimizing the morphology,^12^ their photocatalytic activity as thin films and the influence of the CuWO_4_/WO_3_ ratio on the photocatalytic response were never explored. A comprehensive understanding of these two parameters remains a critical unaddressed novelty.

Additionally, it has been shown that TiO_2_ overlayers^26^ can act as a “leaky” protection layers^26–28^ by allowing photogenerated holes to pass through via tunneling (hopping channels).^26–28^ We tested the effectiveness of TiO_2_ overlayers and proposed that they further improve the photocatalytic activity of the WO_3_/CuWO_4_ heterojunction by acting as a photohole selector and passivating intraband-gap Cu^2+/+^ surface defects that are detrimental to the lifetime of carriers.^26–30^

This work builds on our two previous investigations. In the first,^14^ it was demonstrated that the WO_3_/CuWO_4_ ratio plays a decisive role in determining the photovoltage and photocurrent of type-II photoanodes, allowing the identification of an optimal compositional range that maximizes charge separation and photoelectrochemical efficiency. The second^20^ one introduced ultrathin TiO_2_ overlayers deposited via sputtering, which acted as passivation and hole-selective layers, suppressing surface recombination and enhancing the electrochemical stability of the photoelectrodes. The present study integrates and expands upon these two approaches by combining the compositional optimization of WO_3_/CuWO_4_ heterojunctions with a TiO_2_ surface overlayer while shifting the focus from photoelectrochemical water oxidation to photocatalytic degradation of organic pollutants under visible light. Unlike previous contributions, centered on solar-fuel conversion and oxygen evolution, this report emphasizes the synergistic role of stoichiometric control and surface passivation from an environmental perspective.

Therefore, to optimize the WO_3_/CuWO_4_ ratio aiming at the photocatalytic performance and apply the TiO_2_ overlayers onto it, a set of WO_3_/CuWO_4_ was deposited by reactive magnetron co-sputtering, followed by 600 °C annealing in air. The samples were studied by X-ray diffraction (XRD), Rietveld refinement, Raman spectroscopy, scanning electron microscopy (SEM), atomic force microscopy (AFM), and optical measurements and analyzed by their photocatalytic activity as a function of the composition. Through photocatalytic tests, it was confirmed that efficiency is controlled by the WO_3_/CuWO_4_ ratio, and their photocatalytic response was greater than those of pure WO_3_ and CuO samples, which is a result of the type-II heterojunction. A WO_3_/CuWO_4_/TiO_2_ sample demonstrated the best photocatalytic performance among all WO_3_/CuWO_4_ responses due to TiO_2_ acting as a hole-selective overlayer. The detailed discussion is presented in the next sections.

## Experimental Section

### Preparation of the WO_3_/CuWO_4_ Samples

FTO/glass (15 Ω/sq) and silica glass substrates from MSE were used to deposit the samples. Both substrates were cleaned in ultrasonic baths (10 min in deionized water and neutral detergent Merck Extra MA 02, followed by 10 min in acetone (Synth 99.5%), 10 min in isopropyl alcohol (Merch 99.8%), and finally dried in hot air. The tungsten target (99.999%) and the copper target (99.99%) were bought from Macashew—Brazil and Kurt J. Lesker Company—USA, respectively, and the gases, Ar and O_2_, 99.999%, both of 5N purity, were provided by the White Martins Company—Brazil.

A Kurt J. Lesker commercial Reactive RF and DC magnetron co-sputtering system was used to grow the samples. In the RF source (RFX600), 180 W of constant discharge power was delivered at the W target, while a different discharge power of the DC source (MDX500) was used in the Cu target (1.0, 2.0, 3.0, 4.0, and 5.0 W). The two sputtering targets were positioned at an angle of 45°, with the substrate holder located between them. The target-to-substrate distance, measured from the center of each target to the center of the substrate holder, was fixed at 10 cm.^14^ The pressure of 5 × 10^–3^ Torr, and the mixture of Ar and O_2_ gases with flow rates of 30 and 10 sccm, respectively, were kept constant during all depositions. The temperature in the substrate surface, measured by an attached thin cromel–alumel thermocouple, was about 300 ± 20 °C, even though the temperature in the Neocera heater/controller was 500 °C. This temperature difference is caused by losses in the heater–substrate interface, by the low thermal conductivity of glass, and by the room temperature chamber walls.^31^ After the deposition time, all samples were annealed in air at 600 °C for 2 h using an *EDG-3PS* box furnace. The annealing process was performed by gradually increasing the temperature at a rate of 1 °C/min until reaching 600 °C. To complete the process, samples were cooled naturally from 600 to 25 °C over the course of ∼7 h.

### Preparation of the WO_3_/CuWO_4_/TiO_2_ Samples

TiO_2_ depositions over WO_3_/CuWO_4_ samples were conducted by applying 240 W on the RF power source using a metallic Ti Kurt J. Lesker target (99.999%), positioned 8 cm from the substrate holder. Ar and O_2_ gases at flow rates of 40 and 2 sccm, respectively, were inserted into the deposition chamber, and 5 × 10^–3^ Torr of total pressure is used during the deposition. The temperature during the deposition was controlled by a Neocera heater/controller adjusted to 250 °C, but the real temperature was 150 ± 20 °C due to losses at the interface of the heater/substrate holder and substrate holder/samples. Moreover, none of the samples were annealed after these depositions.

### Characterization

XRD experiments were performed in a PANalytical Empyrean diffractometer using Cukα (λ = 1.540 60 Å) radiation, using a Bragg–Brentano θ–2θ configuration, with a 0.01° step size in the 10°–60° range, with Rietveld analysis performed with GSAS software. The Raman spectra were acquired using a LabRAM Odyssey Raman spectrometer equipped with an excitation laser at 532 nm. Spectral acquisition was performed in the 100–1000 cm^–1^ range. A 100x long working distance objective was employed for the measurements.

Morphological characterizations were performed via SEM and AFM. SEM measurements were obtained with a JEOL microscope, model JSM-IT500HR, equipped with secondary electron (SE), backscattered electron (BSE) detectors, and chemical analysis (X-ray energy-dispersive spectroscopy—XEDS). AFM measurements were performed with an atomic force microscope Park XE7. Images in noncontact mode, with an area of 5 × 5 μm, were acquired to derive the roughness factor (RQ), which indicates the root-mean-square (RMS) of the samples.

Optical transmittance and absorbance measurements were performed with a PerkinElmer spectrophotometer (Lambda 1050 UV/vis/NIR) with a data interval of 1.00 nm (200–1800 nm range) and a scan speed of 141 nm/min.

Surface photovoltage spectroscopy was performed in an air environment utilizing a semitransparent vibrating gold-mesh Kelvin probe (Delta PHI Besocke) controlled by a Kelvin Control 7 Oscillator/amplifier (Besocke Delta Phi). The TiO_2_ sample was illuminated through the Kelvin probe with monochromatic light in the 3390–40,000 cm^–1^ range generated by an Oriel Cornerstone 130 monochromator powered by a 300 W Xe arc lamp source with 50–150 μW/cm^2^ light intensity. Spectra were obtained by increasing steps of 0.0124 eV of the photon energy every 5 s and by collecting the contact potential difference (CPD) value at each step. The SPV signal was acquired according to the following equation:

$$\mathrm{SPV}={\mathrm{CPD}}_{\mathrm{light}}-{\mathrm{CPD}}_{\mathrm{dark}}$$ 1

Incident photon-to-current efficiency (IPCE) characterization was performed using a Gamry Reference 600 Potentiostat linked to a photoelectrochemical cell (PEC), filled with 50 mL of 0.1 M Na_2_HPO_4_/NaH_2_PO_4_ buffer solution at pH 6.5, constantly stirred at 300 rpm. The PEC has a three-electrode setup (Pt as a counter electrode, a saturated calomel reference electrode separated by a KCl salt bridge, and the working electrode). It was performed at 1.23 V vs RHE (calibrated using the redox potential K_4_[Fe­(CN)_6_] at 0.358 V (NHE)) using light from a monochromator from Instruments SA Inc. H10 1200 (350–800 nm) behind a 300 W xenon lamp under AM 1.5 G irradiation (100 mW cm^–2^ under commercial silicon solar cell calibration). The wavelength was varied in 25 nm increments, starting from 875 nm and finishing at 300 nm. By using a NIST traceable radiometer/photometer from *International Light*, we measured the irradiance at each wavelength. Next, IPCE values were calculated using eq 2,^14^ where, *j*_ph_ is the photocurrent density in mA/cm^2^ at 1.23 V RHE, *h* is the Planck constant (6.62 × 10^–34^ J·s), *c* is the speed of light (3.0 × 10^8^ m/s), *e* is the charge of a single electron (1.6 × 10^–19^ C), *P*_mono_ is the power density of monochromatic light in mW·cm^2^, and λ is the monochromatic wavelength of light in nm.

$$\mathrm{IPCE}=\frac{j_{\mathrm{ph}}\times \frac{hc}{e}}{P_{\mathrm{mono}}\times \lambda }\times 100\%$$ 2

### Photocatalytic Tests

The photocatalytic tests were performed in a home-built chamber using a monochromatic blue LED irradiation (405 nm). A thermocirculator was used to refrigerate the external walls of the reactors (∼6 mL internal volume) and maintain the solution temperature at 20 ± 1 °C. Before each photocatalytic test was started, magnetic stirrers were inserted at the bottom of the reactors, and samples were placed 5 mm below the surface. After that, 5.0 mL of methylene blue (MB) aqueous solution containing 2.5 ppm was inserted into each reactor, and the system was left for 120 min in the dark to stabilize adsorption processes. Afterward, 2 mL of the MB solution was collected from each reactor, and its absorbance was measured. These collected samples represented the “initial rate” and were returned to their respective reactors. Lights, which stayed 5 cm away from the reactors, were then turned on, and the photodegradation reaction was started. During the photocatalytic test, which lasts 300 min, the solutions were collected every 30 min. The collection processes were always performed by turning the lamps off when the solution was collected and turning them on again when the solution was returned. Absorbance measurements were recorded in the 750–450 nm range, and the degradation was calculated using eq 3, where *A*_i_ is the initial absorbance and *A_t_* is the absorbance at *t* time, both subtracted from the deionized water absorbance.

$$\mathrm{Degradation}(\%)=\frac{A_{i}-A_{t}}{A_{i}}\times 100$$ 3

## Results and Discussion

The results were divided into two parts: (i) corresponding to single heterojunctions involving copper and tungsten oxides and (ii) heterojunctions where the TiO_2_ overlayer was added.

1. **CuWO**_**4**_ **/WO**_**3**_ **compositional series:** CuWO_4_/WO_3_ samples (∼1.5 × 1.5 cm^2^) were cosputtered on FTO and SiO_2_ substrates by using two targets (tungsten and copper) working simultaneously. The deposition schemes and the respective reactions were published by the author in a previous paper^14^ ^,^ ^20^. Figure 1 represents, in a simplified way, the different stoichiometric samples obtained in the *x*-axis during the five depositions, that is, different distances from the targets brought different materials. Figure S1 shows an image of the inside part of the deposition chamber while the deposition was occurring. Figure S2 shows 25 samples considering each one of the five separate depositions (1, 2, 3, 4, and 5 W of Cu target power supply) on the FTO substrate (set of samples on the SiO_2_ substrate not shown).

**Figure 1:**
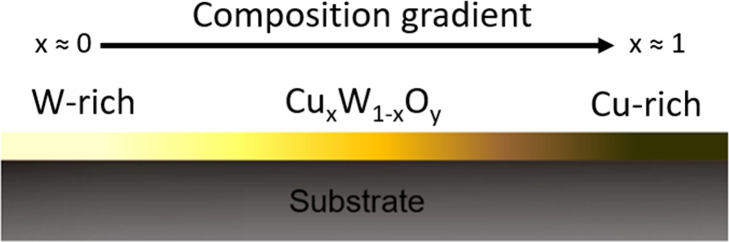
Schematic representation of the combinatorial composition gradient obtained by co-sputtering deposition. The film composition is described as Cu_*x*_W_1–*x*_O_*y*_, where the copper fraction (*x*) increases along the substrate from the W-rich region (*x* ≈ 0) to the Cu-rich region (*x* ≈ 1).

Eight out of the 25 samples were chosen to cover the entire range of different composition materials. The samples were chosen based on similar thicknesses and different band gaps (samples highlighted in Figure S1), the transparent-like samples being closer to the tungsten target, the yellowish samples in the middle, and the brownish samples when closer to the copper target, as shown in Figure 2, due to the WO_3_ band gap being around 2.7 eV,^8^ while the band gap of CuWO_4_ was in the range of 2.2–2.3 eV^8,32^ and that of CuO at 1.2–2.1 eV.^33,34^

**Figure 2:**
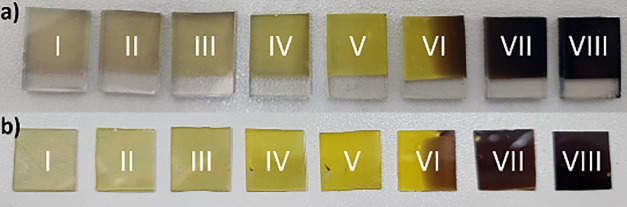
Images of the samples after co-sputtering and thermal annealing at 600 °C. (a) Samples on the FTO substrate. (b) Samples on SiO_2_ substrates. Samples are numerically labeled from I to VIII, where I is closest to the W target and VIII is closest to the Cu target.

To support the expected color evolution, the semiquantitative EDS data exhibited in Table S1 were used to evaluate compositional trends across the sample series. The results indicate a relative increase in the Cu signal intensity accompanied by a decrease in W and O signals, consistent with compositional changes associated with the reduced WO_3_ contribution and increased Cu phases. To accurately determine the crystalline structure and phase evolution, XRD measurements were subsequently performed (Figure 3). Peaks were identified and indexed from the following profiles: WO_3_ in its triclinic structure (JCPDS 71-0305), space group *P*-1; CuWO_4_ also in its triclinic structure and space group *P*-1 (JCPDS 88-0269); tetragonal SnO_2_ peaks, space group *P*42/*mnm*, from the FTO substrate (JCPDS 71-0625); and monoclinic CuO peaks, space group *C*1*c*1 (JCPDS 80-0076). The CuO phase appears exclusively at the copper-rich compositional limit (sample VIII) and is therefore considered a natural outcome of the co-sputtering process under high Cu content. It is not a targeted heterostructure within the scope of this study, but it is briefly discussed for the sake of completeness. The pure WO_3_ sample in Figure 3a,b and the pure CuO in Figure 3e,f were used as references to compare the diffractograms. The amount and intensity of WO_3_ peaks were getting lower, from samples I to VI (Figure 3a–f); in contrast, the CuWO_4_ peaks increased from samples I to VI. Samples VII (Figure 3e,f) displayed only CuWO_4_ peaks, and samples VIII were the only two presenting CuO peaks. Overall, diffractograms of the samples on FTO substrates exhibited less intense peaks but a higher quantity of them when compared to the samples’ diffractograms on SiO_2_ substrates, as the FTO substrate is crystalline and exhibits higher surface roughness than the SiO_2_ substrate. Besides, more preferential peaks can be observed in the SiO_2_ substrate diffractograms, as SiO_2_ is amorphous and exhibits lower roughness, and there is a tendency to grow fewer but more intense planes (minimizing surface energy, affecting nucleation and growth). Furthermore, sputtering deposition is well-known to produce preferentially oriented thin films with a strong texture, which can modify relative peak intensities compared to powder diffraction standards reported in the literature.^35–37^ Such a preferential orientation affects mainly the intensity distribution of diffraction peaks rather than phase identification. Therefore, the reference diffraction patterns were used for phase identification, while differences in relative peak intensities are attributed to texture effects commonly observed in sputtered thin films.

**Figure 3:**
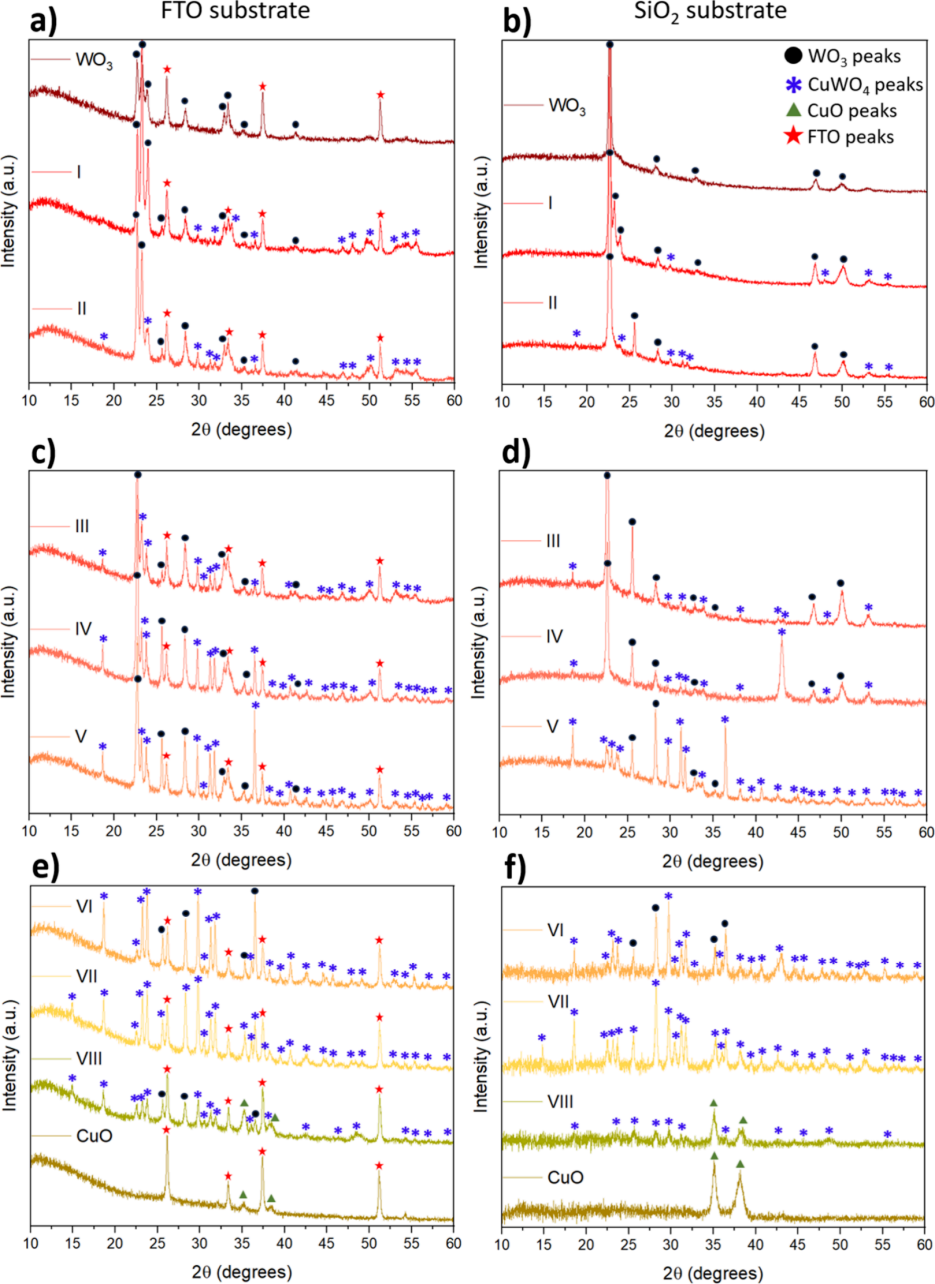
XRD diffraction patterns for the samples. (a, c, and e) Samples deposited on FTO substrate. (b, d, and f) Samples deposited on SiO_2_ substrate. The different materials’ peaks legends are present in (b).

Additionally, because the WO_3_/CuWO_4_ set of films was annealed at 600 °C on FTO substrates, limited diffusion of Sn from the conductive oxide into the deposited layer cannot be completely excluded. However, no Sn secondary phases were detected by XRD, suggesting that any diffusion is restricted to a thin interfacial region below the detection limit of bulk techniques. Since photocatalysis is, predominantly, a surface-driven phenomenon occurring at the semiconductor/solution interface, any potential Sn diffusion would remain localized near the buried substrate interface and is, therefore, considered negligible.

Next, to confirm the XRD analysis, Rietveld Refinement^38^ calculations were performed using the aforementioned crystalline structures and their respective space groups as model inputs. Table 1 reveals the phase atomic percentage of each sample and its respective χ^2^ (a measure of the adjustment quality). The results corroborate with the color trend analysis and XRD peak identification since the WO_3_ percentage tends to decrease from I to VI, while the CuWO_4_ percentage tends to increase for the same samples.

**Table 1:** Rietveld Refinement Calculation Obtained from Samples Deposited on the FTO Substrate in Atom (%)

samples	WO_3_ (%)	CuWO_4_ (%)	CuO (%)	SnO_2_ (%)	χ^2^
I	73.23	12.07		14.70	3.463
II	61.84	19.70		18.46	3.654
III	57.73	25.29		16.98	3.422
IV	45.57	39.23		15.20	4.601
V	30.19	56.56		13.25	4.525
VI	18.39	63.42		18.19	4.785
VII		80.45		19.55	3.295
VIII		34.36	46.92	18.72	2.910

Moreover, SEM measurements illustrate the WO_3_-rich samples I, II, and III (Figure S3a–c) with grains grown with a preferred direction orientation. Samples IV and V (Figure S3d,e) are composed of small spherical grains surrounded by visible plateaus, identified in the last paper as comprising Cu element.^14^ In Figure S3f, sample VI, the inhomogeneous sample, exhibits a porous structure with elongated and narrow grains. Lastly, samples VII and VIII (Figure S3g,h) exhibit compact random grains. Overall, some of them have a preferred orientation style, and all samples reveal compact surfaces, typical of sputtered films.^35–37^ To complement this, AFM measurements were performed to derive the RMS of the samples (Table 2). Samples on the FTO substrate displayed RMS values primarily influenced by the substrate, with all of them similar. On the other hand, for the samples on the SiO_2_ substrate, there is a volcano-like trend, with the RMS values increasing as the CuWO_4_ content increased in the heterojunction.

**Table 2:** Thicknesses Obtained from the Interference Fringes^39^ and Roughness Obtained from AFM Measurements after Annealing in Air at 600 °C

samples	thickness (nm) on SiO_2_ substrate	RMS (nm) on FTO substrate	RMS (nm) on SiO_2_ substrate	band gap (eV) on FTO substrate	band gap (eV) on SiO_2_ substrate
WO_3_		25.94	2.68		
I	873	25.51	2.31	2.73	2.72
II	888	27.55	3.03	2.64	2.68
III	868	24.85	4.30	2.50	2.61
IV	806	27.11	6.23	2.33	2.24
V	846	25.56	12.66	1.99	1.96
VI	788	28.24	11.55	1.87	1.67
VII	775	25.57	6.65	1.59	1.47
VIII	832	26.90	12.04	1.01	1.19
CuO		22.88	2.33		

To further explore the structural properties, Raman spectroscopy measurements reveal information on the vibrational lattice modes of the samples. Due to the similarity in the spectra between both substrates, since the Raman measurements detect the internal vibration from the atomic structure, not the orientation by itself, only the spectra obtained from the samples deposited on SiO_2_ substrates are shown in Figure 4. WO_3_ vibrations (O–W–O and W–O–W)^40,41^ were found for pure WO_3_ and for samples I–VI (Figure 4a–c) around 802, 698, 320, 266 cm^–1^, while CuWO_4_ vibrational modes increased from sample I to VI. The main 903.2 cm^–1^ peak attributed to the symmetric W–O stretching increased and other vibrational modes (antisymmetric W–O vibrations of the WO_6_ clusters at 776.8, 723.4, 673.6, 545.9, 474.5, and 394.4 cm^–1^ and the motion of the WO_6_ clusters against Cu that correspond to the bending, translational, or rotational modes at 353.2, 310.4, 278.2, 219.6, and 187.2 cm^–1^) appeared as the CuWO_4_ content increased.^42^ Sample VII (Figure 4c) displayed only CuWO_4_ vibrational modes, following the XRD results. Sample VIII was the only one in which CuO vibrational modes could be seen, as the pure CuO sample (Figure 4c) exhibits vibrational modes at 631, 346, and 296 cm^–1^.^43–46^ Overall, all of the spectra matched the XRD results.

**Figure 4:**
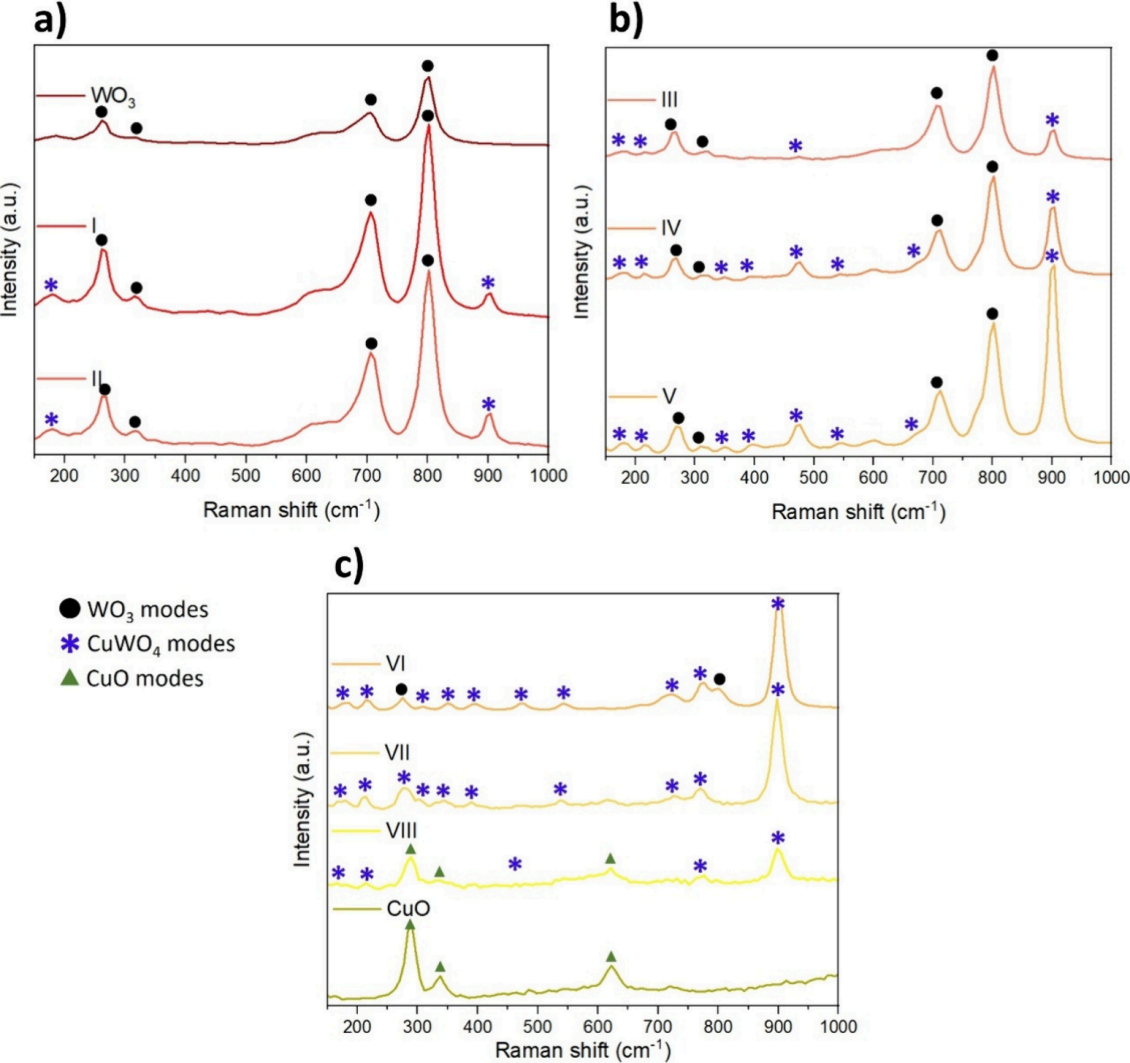
Raman spectra of WO_3_, I, and II (a), III, IV, and V (b), and VI, VII, VIII, and CuO (c). All spectra are those of the samples deposited on a SiO_2_ substrate.

Optical transmittance spectra are shown in Figure 5a,b, corresponding to the heterostructures deposited onto FTO and silica glass substrates (SiO_2_), respectively. A trend is observed across the spectra: as more CuWO_4_ and CuO are incorporated into the samples, the absorption edge shifts toward higher wavelengths. This behavior is consistent with the bandgap values reported for WO_3_ (2.7 eV),^8^ CuWO_4_ (2.2–2.3 eV),^8,32^ and CuO (1.2–2.2 eV).^33,34^ To quantify the effect, Tauc plots were used, and the optical band gaps were estimated from them, assuming that the materials are homogeneous. The slightly lower bandgap values observed for some samples can be attributed to optical effects associated with the presence of multiple interfaces within the heterostructured films. These factors may promote light scattering and internal reflections, modifying the absorption edge and leading to an apparent red shift in the Tauc-derived band gap.

**Figure 5:**
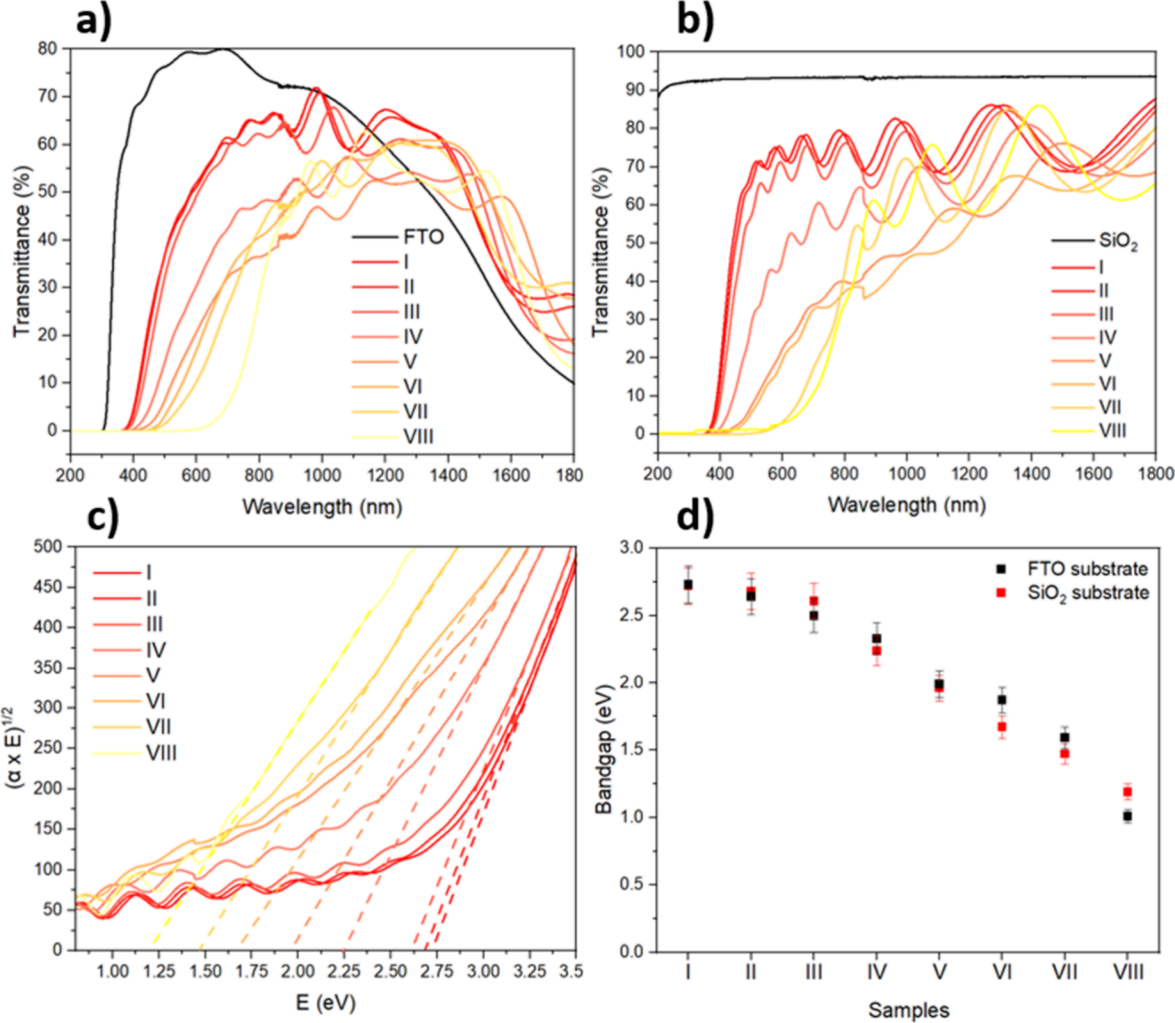
Optical transmittance spectra and optical band gap of the WO_3_/CuWO_4_ samples. Transmittance of samples deposited on the (a) FTO substrate and (b) SiO_2_ substrate. (c) Optical band gap obtained from the Tauc plot method. (d) Band gap comparison between samples deposited on FTO and SiO_2_ substrates with 5% error bars.

The Tauc plots and the values of the corresponding band gaps are displayed in Figure 5c,d and Table 2, respectively. The results support the idea of narrowing the band gap by increasing CuWO_4_ and CuO and decreasing the WO_3_ content. Additionally, from the interference fringes of the transmittance spectra, the thickness was estimated, also considering the homogeneity of the samples.^39^ Table 2 presents values around 800–900 nm, and the SEM cross-sectional measurement in sample V deposited on the FTO substrate in Figure S4 confirmed it.

As the thicknesses are similar but the roughness is not, the drop in the overall transmittance as CuWO_4_ increased, from I to VI (Figure 5b), could be associated with the increase of the interfaces and the consequent light scattering, as well as the presence of Cu^2+/+^ defect states.^19^ As previously described by Wu et al.,^19^ these CuWO_4_ defects also contribute to the broadening of the absorption edge. This behavior is particularly evident in samples V and VI, especially with samples on the SiO_2_ substrate, as suggested by their transmittance spectra in Figure 5b. In contrast, sample VII, identified as pure CuWO_4_, does not exhibit a significantly broadened absorption edge compared to samples V and VI, although its band gap is shifted to lower values (1.59 eV on FTO and 1.47 eV on SiO_2_). Furthermore, the narrowing bandgap phenomenon could also be occurring for previously mentioned samples, V and VI. This suggests that Cu^2+/+^ defect states are being suppressed during deposition, possibly due to the incorporation of additional Cu into the CuWO_4_ crystal structure, or due to the presence of trace amounts of CuO in the samples, which are undetectable by XRD and not evidenced in Raman measurements, but still contribute to narrowing the band gap.

Even though there are differences in bandgap values between both substrates, evidenced in Table 2 and Figure 5d, they follow the same overall trend with composition, indicating that the observed band gap narrowing is intrinsic to the films rather than substrate-dependent.

On the photocatalytic tests, MB was used as a dye model due to the impossibility of degrading a series of emergent organic waste compounds, such as bisphenol A,^47^ ibuprofen,^48^ dipyrone,^49^ and acetaminophen.^50^ MB is cationic, which indicates that the adsorption of the dye is favorable onto the photocatalyst surface, while the others are neutral/anionic, unfavorable for adsorption.^51,52^ Adsorption tests revealed a significant absorbance loss in the chromophoric peak of the MB solution during the first hour of the test, while no significant absorbance value changes were observed for the waste organic compounds, confirming the adsorption hypothesis. A representative adsorption experiment before irradiation, obtained for sample V deposited on SiO_2_, is presented in the Supporting Information (Figure S5).

The photocatalytic activity toward MB is likely associated with its efficient adsorption onto the semiconductor surface, which promotes close proximity to active sites and facilitates rapid interaction with photogenerated charge carriers, leading to MB degradation.^51,53^ It was not the case of the waste organic compounds. Additionally, the limited adsorption of neutral/anion materials onto the WO_3_/CuWO_4_ surface probably would require more ·OH radicals for degradation. However, in those compact sputtered thin films, the radical yield per unit volume would be too low and the kinetics would be too slow to achieve detectable removal within short irradiation times during the photocatalytic tests. Thus, the photocatalytic activity of the samples degrading MB aqueous solution, including the pure materials, such as WO_3_ and CuO, is displayed in Figure 6a,b.

**Figure 6:**
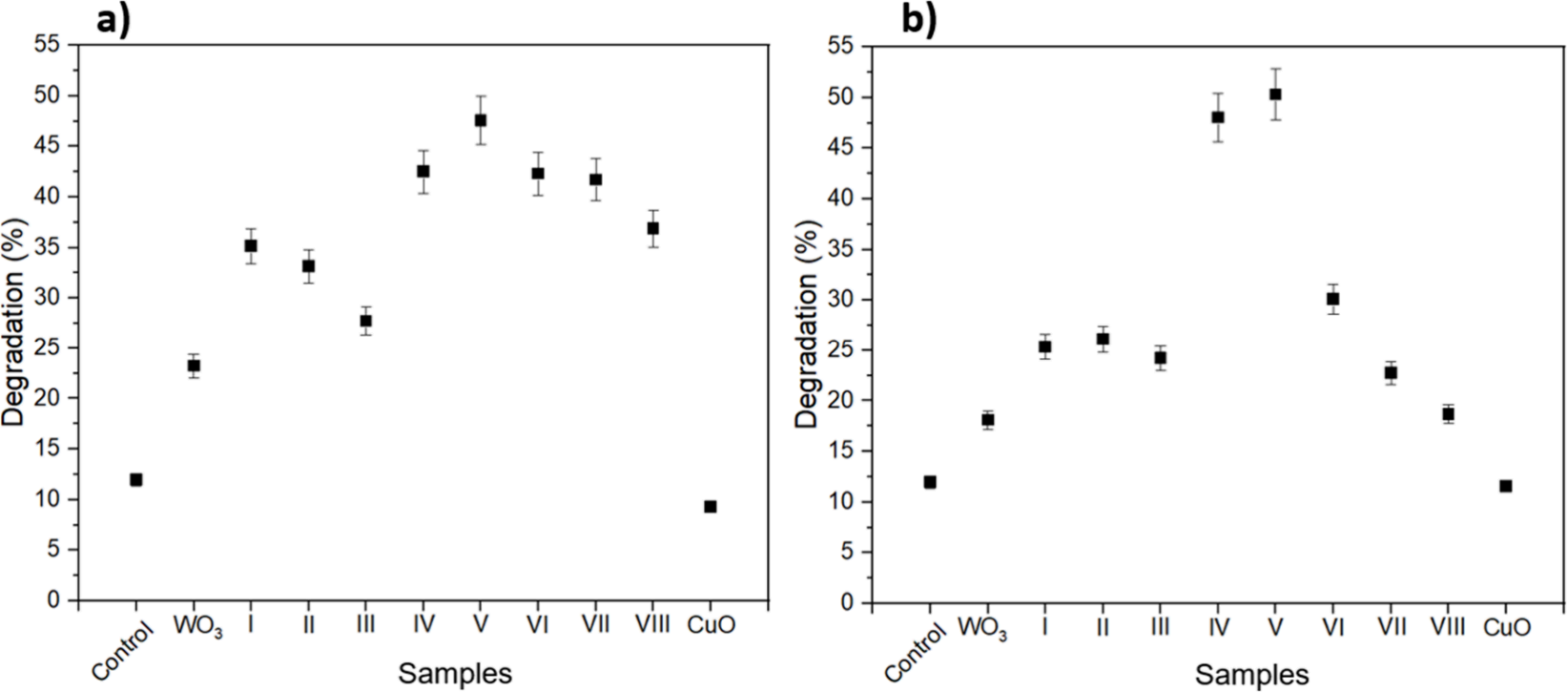
Photocatalytic activity of the samples on the (a) FTO substrate and (b) SiO_2_ substrate. All of the tests were performed using a 405 nm monochromatic LED and lasted 3 h.

The photocatalytic response was obtained with a 405 nm monochromatic LED during 300 min of light irradiation. Each sample was subjected to three photocatalytic test cycles, and no losses of efficiency were detected after the third one. Table S2 summarizes the stability and recyclability results for the samples exhibiting the highest photocatalytic performance (IV and V) deposited on FTO and SiO_2_ substrates.

Pure WO_3_ and CuO demonstrated less photocatalytic activity than the overall heterojunctions, which was expected since they facilitate charge carrier separation, enhancing photoactivity.^8,12^ Results shown in Figure 6a,b suggest there is a maximum percentage efficiency performance between WO_3_ and CuWO_4_ phases, since samples IV (45.57% WO_3_ and 39.23% CuWO_4_) and samples V (30.19% WO_3_ and 56.56% CuWO_4_) were revealed to have better photocatalytic response than the others. The following hypothesis can be explored: AFM measurements (Table 2) show no considerable change in the RMS roughness for samples on the FTO substrate that justifies the best performance. In contrast, qualitative inspection of the SEM images (Figure S3d,e) reveals visibly finer surface features, suggesting an increased WO_3_/CuWO_4_ heterojunction contact that may contribute to the observed photocatalytic response.

Moreover, while the hole diffusion length in WO_3_ is 4–6 times higher than that in CuWO_4_, the electron–hole pair generation rate exceeds that of WO_3_.^12,54–57^ The higher heterojunction interface area and the stronger carrier generation in CuWO_4_, combined with a longer hole-transport length in WO_3_, provide a coherent explanation for the superior heterojunction performance.^12,54–57^ In contrast, samples IV and V on SiO_2_ in Figure 6b doubled the photocatalytic efficiency when compared to the others. This can be explained by the higher heterojunction charge separation efficiencies but also the increase in RMS with an increase in the CuWO_4_ content in the samples. For low-CuWO_4_-content samples (I, II, and III), the performance decreased, as compared to those of IV and V, due to the decreased heterojunction interface area. Besides, since sample VII only presented CuWO_4_, there is no heterojunction to facilitate the separation of charges.

Samples I–VII can only carry out oxidative photocatalysis due to the WO_3_ and CuWO_4_ conduction band positions (not enough energy to generate ·O_2_^–^), as illustrated in Figure 7a, limiting the reaction by the interaction with the ·OH radicals.^58^ Finally, sample VIII, composed of CuWO_4_ and CuO, exhibits oxidative photocatalytic activity primarily on the CuWO_4_ surface, while reductive reactions are favored on the CuO surface. However, this combination does not result in optimal performance as the band bending between the semiconductors (Figure 7b) promotes electron transfer toward the CuWO_4_ interface, competing with electrons that would otherwise reach the surface.

**Figure 7:**
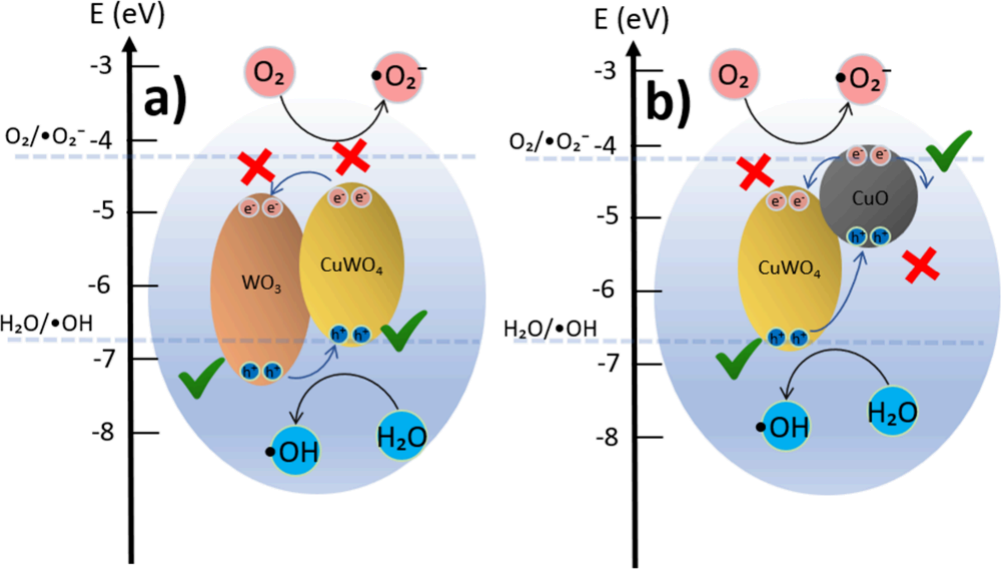
Energy diagrams and charge transfer in (a) WO_3_/CuWO_4_ and (b) CuWO_4_/CuO. The schemes illustrate the oxidative and reductive photocatalysis of the materials, and the band edge positions were taken from the literature.^12,54,59^ The red crosses indicate where oxidative or reductive photocatalytic reactions are not allowed, while the green check marks denote energetically allowed photocatalytic pathways.

Finally, to compare the photocatalytic behavior across the compositional series, the degradation kinetics were analyzed using an apparent pseudo-first-order (PFO) model.^60^ Deviations from ideal PFO behavior during the initial irradiation period (0–30 min) are attributed to transient adsorption–desorption processes, surface equilibration, and carrier trapping dynamics typically observed in thin-film photocatalysts. After this transient regime, the system tends to reach a quasi-steady-state condition in which the degradation kinetics are better described by a PFO model (Figures S7 and S8). For this reason, the kinetic constants were obtained from the linear regime of ln­(*C*_0_/*C*) within the 30–300 min range.

The extracted apparent rate constants (*k*) and corresponding *R* ^2^ values are summarized in Tables 3 and 4. For samples deposited onto FTO, intermediate compositions (IV and V) exhibited the highest k values, consistent with the degradation trends shown in Figure 6a. A similar behavior was also observed for samples deposited on the SiO_2_ substrate (Figure 6b), where compositions IV and V also presented the fastest kinetics. The high *R* ^2^ values (≥0.97 for most samples) confirm that the PFO model adequately describes the dominant kinetic regime.

**Table 3:** Apparent PFO Rate Constants (*k*, min^–1^) and Corresponding *R* ^2^ Values Obtained from Linear Fits of the Degradation Kinetics for Samples on FTO Substrates

	control	I	II	III	IV	V	VI	VII	VIII
*k* (min^–1^)	0.0004	0.0017	0.0013	0.0011	0.0017	0.0019	0.0016	0.0016	0.0012
*R* ^2^	0.986	0.996	0.981	0.989	0.976	0.968	0.969	0.973	0.975

**Table 4:** Apparent PFO Rate Constants (*k*, min^–1^) and Corresponding *R* ^2^ Values Obtained from Linear Fits of the Degradation Kinetics for Samples on SiO_2_ Substrates

	control	I	II	III	IV	V	VI	VII	VIII
*k* (min^–1^)	0.0004	0.0010	0.0009	0.0011	0.0021	0.0024	0.0011	0.0008	0.0006
*R* ^2^	0.985	0.996	0.974	0.995	0.996	0.989	0.973	0.994	0.986

These results reinforce the conclusion that the enhanced photocatalytic performance of intermediate WO_3_/CuWO_4_ compositions arises primarily from improved charge separation at the type-II heterojunction interface combined with favorable surface morphology effects, particularly an increased heterojunction interface area and surface roughness.

**WO**_**3**_ **/CuWO**_**4**_ **/TiO**_**2**_ **set of samples**: To further study the improvement of the WO_3_/CuWO_4_ heterojunction photocatalytic response, TiO_2_ surface overlayers of variable thickness were deposited by RF magnetron sputtering.^6^ The TiO_2_ thickness was controlled via the deposition time. An 8-h deposition was conducted, and 196 nm of TiO_2_ was obtained.^39^ Next, deposition with controlled time was performed to obtain 2, 4, 8, 16, 48, 64, 96, and 128 nm of TiO_2_ overlayers. SEM cross-section measurement (Figure S6) supports the idea of controlling the thickness by deposition time since the sample thickness calculated at 128 nm of TiO_2_ overlayer by deposition time matches well with the observed results. This set of samples was previously characterized via photoelectrochemistry measurements and water splitting tests to generate O_2_, but they were not applied to photocatalytic tests.^20^ A schematic showing the samples having CuWO_4_/WO_3_, CuWO_4_/TiO_2_, and WO_3_/TiO_2_ interfaces can be seen in the previous author’s published paper.^20^ The previous results demonstrated a photoresponse improvement in some of them (2, 4, and 8 nm of TiO_2_ overlayer).^20^ It was attributed to the passivation of Cu^2+/+^ surfaces defects of CuWO_4_ ^19^ and the selectivity of the TiO_2_ for photoholes.^28,61^ Thick TiO_2_ overlayers displayed decreased photocurrent and photovoltage, revealing an increased hole-transfer resistance and light shading effects.^20^ In this way, photocatalytic tests under 405 nm LED illumination were performed to evaluate and compare the photoelectrochemistry and photocatalytic results. The WO_3_/CuWO_4_ sample used in this section corresponds to the optimized compositional ratio identified in our previous study. For the present work, the phase composition was estimated by Rietveld refinement, confirming that all samples consist of approximately 60% CuWO_4_ and 40% WO_3_, with or without the TiO_2_ overlayer. Pure TiO_2_ and pure WO_3_/CuWO_4_ were first applied to the photocatalytic test (Figure 8), and their photocatalytic activities (29 and 32%) were revealed. Then, the WO_3_/CuWO_4_/TiO_2_ heterojunctions were subjected to the tests, revealing an increased photocatalytic response in almost all of the samples, with an average activity between 37.5% and 40%, higher than that of the pure samples.

**Figure 8:**
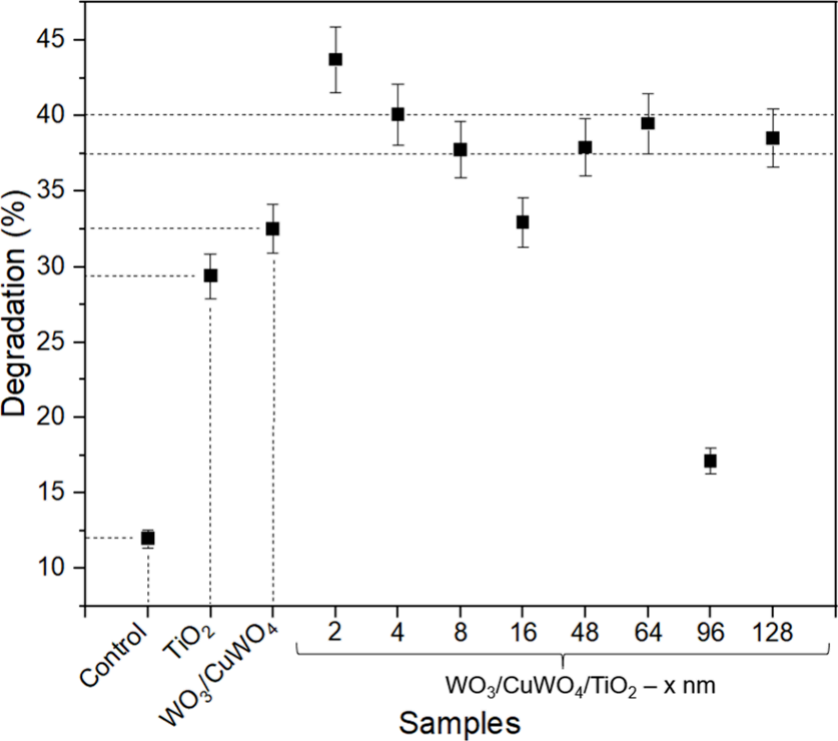
Photocatalytic degradation efficiency of WO_3_/CuWO_4_/TiO_2_ heterostructures under 405 nm LED irradiation for 3 h. Each data point represents the value obtained from independent experiments, and the error bars indicate the standard deviation. The TiO_2_ overlayer thicknesses are shown at the *x* axis (2, 4, 8, 16, 48, 64, 96, and 128 nm). The results are compared with the control sample, pure TiO_2_, and WO_3_/CuWO_4_.

To understand the overall activity, it is necessary to evaluate the TiO_2_ photocatalytic activity first. As a 405 nm monochromatic LED is used to evaluate the photoresponse, the band gap of the materials should be lower than 3.06 eV. In this case, Tauc’s plot (Figure S7) indicates an optical TiO_2_ band gap of 3.05 eV, a value quite similar to that required theoretically. To complement this, vibrating Kelvin probe surface photovoltaic spectroscopy (SPS) was employed. The CPD of the sample was measured with a semitransparent gold Kelvin probe.^62^ The change CPD (based on the gold reference electrode) in going from dark to illumination defined the surface photovoltage signal based on the following formula: SPS = CPD_(light)_ – CPD_(dark)_.^63–65^ The spectrum in Figure 9a confirms a negative SPS signal, typical for N-type semiconductors, such as TiO_2_, and revealed an effective band gap of 2.68 eV, smaller than the optical one. Furthermore, the IPCE measurement in Figure 9b confirms the generation of electron–hole pairs at 405 nm (with applied 1.23 V vs RHE), corroborating the SPV analysis. In short, TiO_2_ is capable of photocatalysis with 405 nm LED illumination, even though its light absorption is not as prominent at this wavelength.

**Figure 9:**
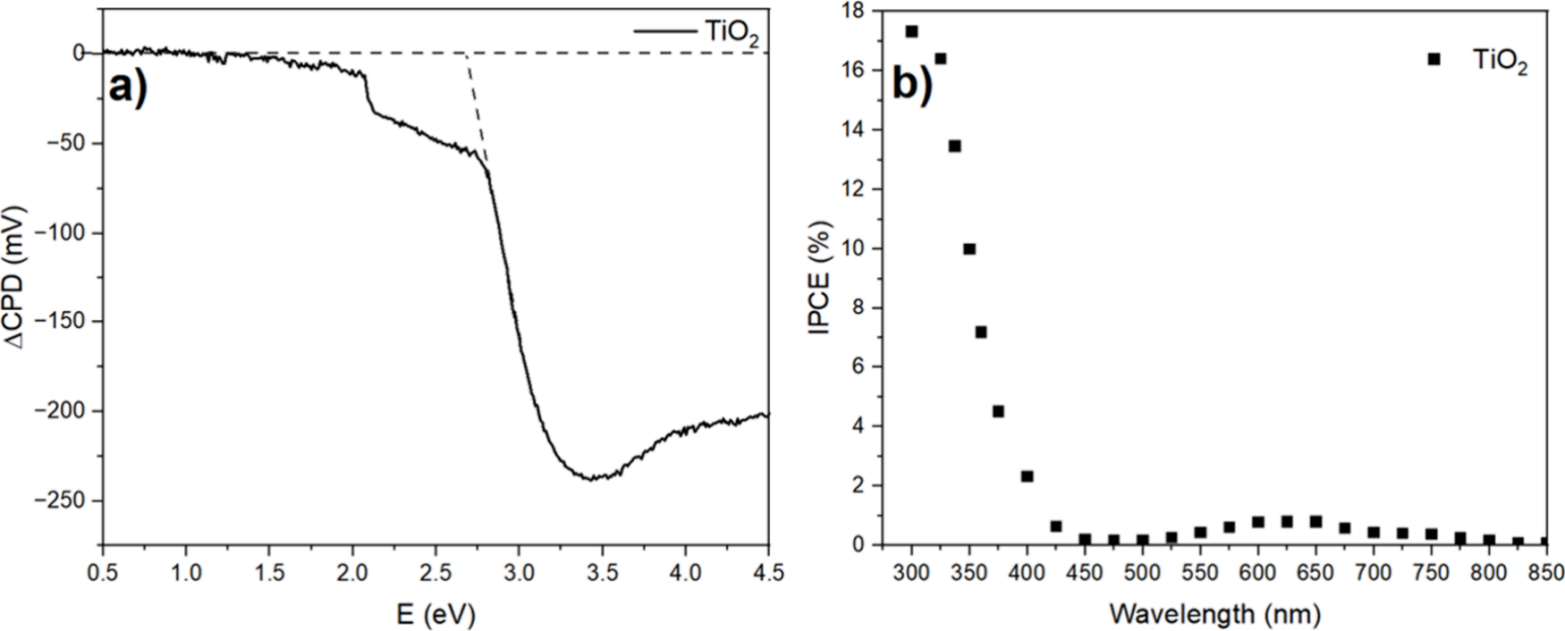
(a) Vibrating Kelvin probe surface photovoltage spectra recorded on the TiO_2_ sample in air. (b) Photoaction spectra for the TiO_2_ sample in 0.1 M phosphate buffer solution at pH 6.5. Nonzero IPCE values at λ > 550 nm are a measurement artifact because of the second-order diffraction from the monochromator optical grating.^20^

Next, analyzing the other samples, WO_3_/CuWO_4_/TiO_2_ (2 nm) degraded 44% of the MB aqueous solution during the 300 min of photocatalytic test, ∼20% more efficient than the pure material sample, an exception among the samples. Since photocatalysis is a surface phenomenon, the heterojunction effects and the hole transport in the “leaky” amorphous TiO_2_ ^28,61^ may increase the separation of charges, as displayed in Figure 10a. The holes generated at WO_3_ and CuWO_4_, near the WO_3_/TiO_2_ and CuWO_4_/TiO_2_ interface, could reach the TiO_2_ surface via hopping channels.^28,61^ Besides, TiO_2_ can perform reductive photocatalysis by absorbing light and generating the electron–hole pair by itself, since its conduction band has the required energy to produce superoxide radicals,^58^ enhancing the photocatalytic activity.

**Figure 10:**
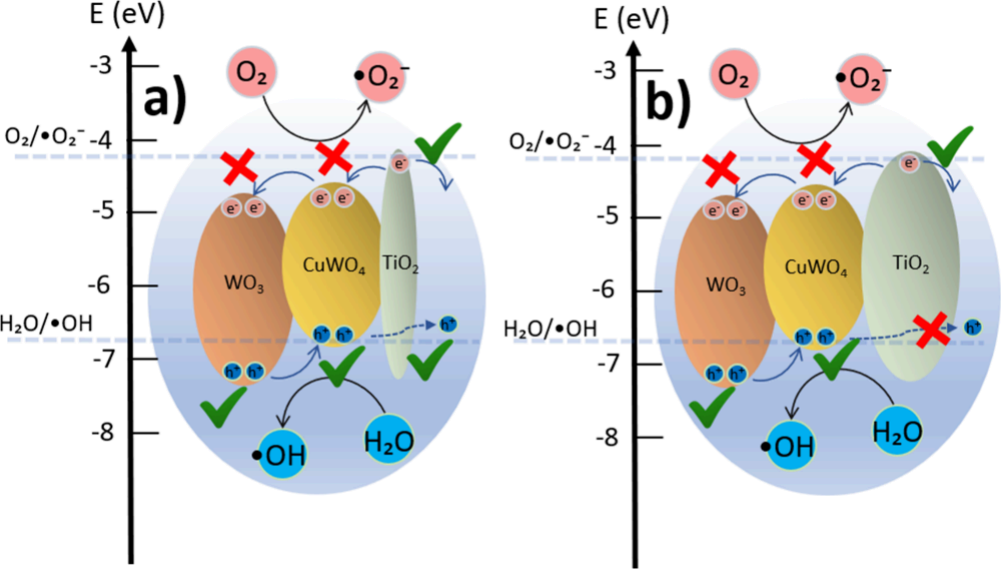
Energy diagrams and charge transfer in (a) WO_3_/CuWO_4_/TiO_2_ (2 nm) and (b) WO_3_/CuWO_4_/TiO_2_ with thicker overlayers. The schemes illustrate the oxidative and reductive photocatalysis of the materials, and the band edge positions were obtained from the literature.^12,54,59^ The red crosses indicate where oxidative or reductive photocatalytic reactions are not allowed, while the green check marks denote energetically allowed photocatalytic pathways.

When the TiO_2_ overlayer becomes thick, the “leaky” effect of TiO_2_ becomes negligible, like the photocatalysis results from WO_3_/CuWO_4_/TiO_2_ (4, 8, 16, 48, 64, 96, and 128 nm) displayed in Figure 8. The same phenomenon was observed in a previous paper.^20^ Therein, 8 nm of TiO_2_ overlayer was enough for the “leaky” effect to start decreasing. In that case, photoelectrochemical measurements were performed by linear sweep voltammetry (0.4 to 1.6 V vs RHE). Now, as there is no potential applied and only the photocatalytic properties are being evaluated, the “leaky” TiO_2_ effect was observed only for the sample with the thinnest TiO_2_ overlayer (2 nm).

The photocatalytic results in other samples suggest that only the TiO_2_ overlayer contributes to the photocatalytic response. The sample with a 96 nm TiO_2_ overlayer exhibited a significantly lower degradation efficiency (∼18%) compared to the other TiO_2_ thicknesses, which clustered around 38–40%. At present, the origin of this deviation is not fully understood. Possible explanations would include local film defects, changes in TiO_2_ microstructure or stoichiometry leading to enhanced bulk recombination, or a reduced effective surface area for dye adsorption, but the same phenomenon did not happen to the sample with 128 nm of TiO_2_ overlayer. A more detailed microstructural investigation of this specific sample must be carried out to obtain a conclusive result.

Figure 10b illustrates a possible photocatalytic mechanism for samples with a TiO_2_ overlayer thicker than 2 nm. The WO_3_/TiO_2_ or CuWO_4_/TiO_2_ heterojunction does not create channels for carrier migration between them at the excited states. As the conduction band is more positive and the valence band is more negative, the configuration suppresses the separation of charges at the interface. Furthermore, TiO_2_ becomes too thick to transport holes via hopping channels; as a result, the only possible mechanism is the TiO_2_ absorption of light, generating electron–hole pairs inside the bulk and photocatalysis occurring from the material’s surface. The increase in photocatalytic activity observed when comparing pure TiO_2_ with WO_3_/CuWO_4_/TiO_2_ (thick TiO_2_ overlayer) may be associated with differences in surface roughness. The TiO_2_ overlayers replicate the underlying WO_3_/CuWO_4_ surface morphology rather than inducing significant morphological restructuring, resulting in nearly unchanged RMS values despite variations in the TiO_2_ thickness. Pure TiO_2_ films exhibit an RMS roughness of approximately 20 nm, whereas WO_3_/CuWO_4_/TiO_2_ samples present an average RMS of ∼29 nm, corresponding to an increase of about 45%. This higher roughness may enhance the effective surface area and contribute to the improved photocatalytic response (Table S3).

Although recyclability tests were performed only for the WO_3_/CuWO_4_ films demonstrating stable photocatalytic performance over three consecutive cycles without efficiency loss (Table S2), sputtered TiO_2_ thin films are recognized for their excellent chemical and photocatalytic stability under repeated irradiation conditions, as reported in the literature and in previous studies showing stable activity over multiple degradation cycles.^66^ Therefore, the incorporation of a TiO_2_ overlayer is not expected to compromise the structural or photocatalytic stability of the WO_3_/CuWO_4_/TiO_2_ heterostructures. Dedicated long-term cycling tests for the complete heterostructure will be addressed in future investigations.

At last, the apparent PFO kinetic model^60^ (30–300 min interval) was applied to the WO_3_/CuWO_4_/TiO_2_ sample series (Figure S9). Notably, although deviations from ideal PFO behavior during the initial irradiation stage are observed for several samples, including those from the previous sample set, this effect is markedly more pronounced for the 2 nm TiO_2_ overlayer sample. Despite not exhibiting the highest apparent rate constant extracted from the 30–300 min linear regime (Table 5), the 2 nm sample achieves the highest overall degradation efficiency. This behavior indicates a two-stage kinetic response in which ultrathin overlayers promote an accelerated initial degradation stage before the establishment of a steady-state PFO regime. Consequently, the apparent rate constant primarily reflects the steady-state degradation process, while the enhanced early stage activity plays a decisive role in the overall photocatalytic performance.

**Table 5:** Apparent PFO Rate Constants (*k*, min^–1^) and Corresponding *R* ^2^ Values Obtained from Linear Fits of the Degradation Kinetics for WO_3_/CuWO_4_/TiO_2_ Samples

	control	0 nm	2 nm	4 nm	8 nm	16 nm	32 nm	48 nm	64 nm	96 nm	128 nm	TiO_2_
*k* (min^–1^)	0.0004	0.0012	0.0012	0.0013	0.0017	0.0015	0.0019	0.0016	0.0019	0.0007	0.0015	0.0014
*R* ^2^	0.986	0.990	0.983	0.987	0.979	0.974	0.994	0.986	0.988	0.978	0.983	0.998

## Conclusions

This work demonstrates that heterostructured WO_3_/CuWO_4_ and WO_3_/CuWO_4_/TiO_2_ thin films, fabricated by reactive magnetron sputtering, exhibit tunable structural, optical, and photocatalytic properties as a function of composition and overlayer thickness.

The WO_3_/CuWO_4_ heterojunctions showed enhanced photocatalytic activity compared to the single oxides, which was attributed to the increased interfacial contact area and the complementary charge-transport properties: longer hole diffusion length in WO_3_ and higher electron–hole generation rate in CuWO_4_. Intermediate compositions (samples IV and V) provided the highest efficiencies for MB degradation, confirming the synergistic effect of the WO_3_/CuWO_4_ interface. The introduction of an ultrathin TiO_2_ overlayer (∼2 nm) further improved the photocatalytic performance, possibly due to the passivation of Cu^2+/+^ surface defects in the CuWO_4_ surface and the contribution of “leaky” amorphous TiO_2_ to hole transport. For thicker TiO_2_ overlayers, the photocatalytic activity was dominated by TiO_2_ itself rather than by the heterojunction. Furthermore, the superior performance relative to that of pure TiO_2_ was correlated to the increased surface roughness (RMS) of the heterostructure. Overall, the results highlight heterojunction engineering via magnetron sputtering as a promising route to optimize photocatalytic-based materials. Moreover, proper adjustment of the WO_3_/CuWO_4_ ratio and precise control of the TiO_2_ overlayer thickness are decisive factors to maximize the efficiency in contaminant degradation.

Compared with previous reports,^14,20^ the present results consolidate and expand the understanding of WO_3_/CuWO_4_-based heterostructures. The first report has demonstrated that the compositional ratio WO_3_/CuWO_4_ plays a decisive role in determining photovoltage and photocurrent, while the second showed that ultrathin TiO_2_ films deposited by RF sputtering act as efficient passivation and hole-selective layers, suppressing surface recombination defects and enhancing photoelectrode stability. In the present results, these two approaches are successfully combined, confirming that the simultaneous optimization of WO_3_/CuWO_4_ composition and TiO_2_ surface passivation results in a synergistic enhancement of photocatalytic performance under visible light. Moreover, the current work extends the heterojunction strategy to organic pollutant degradation, thereby broadening its applicability from solar-fuel generation to environmental remediation.

## Supplementary Material



## Notes

Co-sputtering system (Figure S1); set of 25 samples deposited on FTO substrate (Figure S2); SEM images (Figures S3 and S4); UV–vis absorption spectra (Figure S5); cross-sectional SEM image of sample WO3/CuWO4/TiO2 (128 nm) (Figure S6); transmittance data (Figure S7); PFO kinetic plots (Figures S8–S10); chemical composition from EDS (Table S1); stability and recyclability results (Table S2); and RMS values from AFM measurements (Table S3) (PDF)

## Abbreviations

XRD: X-ray diffraction
SEM: scanning electron microscopy
AFM: atomic force microscopy
RQ: roughness factor
RMS: root-mean-square
IPCE: incident photon-to-current efficiency
PEC: photoelectrochemical cell
RHE: reversible hydrogen electrode
DC: direct current
RF: radiofrequency
SE: secondary electron
BSE: backscattered electron
XEDS: X-ray energy-dispersive spectroscopy
EDS: energy-dispersive spectroscopy
