# Supplementary material for: Role of Heterojunction Engineering in Sputtered WO3/CuWO4 and WO3/CuWO4/TiO2 Thin Films for Enhanced Photocatalysis
Source: ACS Omega. 2026 Apr 29;11(18):26909–21. doi: 10.1021/acsomega.6c00156 (PMC13176987; doi:10.1021/acsomega.6c00156)
Supplement: Supplementary file 1 [file ao6c00156_si_001.pdf]

Supporting information

The Role of Heterojunction Engineering in Sputtered $WO_3/CuWO_4$ and $WO_3/CuWO_4/TiO_2$ Thin Films for Enhanced Photocatalysis

Lucas Caniati Escaliante[a]*, Nilton Francelosi Azevedo Neto[b], Luiz Felipe Kaezmarek[a] Pedrini, Kleper de Oliveira Rocha[c], Jose Humberto Dias da Silva[a].

[a] *School of Sciences, Graduate Program in Materials Science and Technology – POSMAT, Universidade Estadual Paulista – UNESP, Bauru, São Paulo*, 17033-360, *Brazil.*

[b]*Plasma and processes laboratory, Instituto de Tecnologia Aeronáutica – ITA, São José dos Campos, São Paulo, 12228-900, Brazil*

[c] *School of Sciences, Chemistry Department, Universidade Estadual Paulista – UNESP, Bauru, São Paulo*, 17033-360, *Brazil.*

* *Email: lucas.caniati@unesp.br*

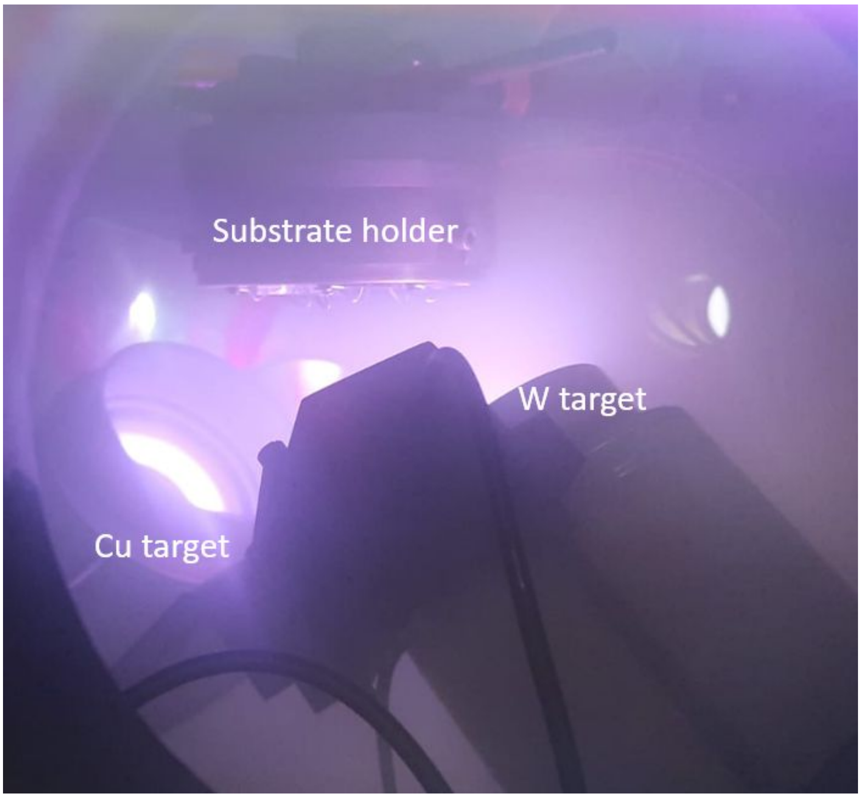

Figure S1. Co-sputtering system with two metal targets. Radio Frequency (RF) discharges are used in the W target, while direct current (DC) discharges of variable power are applied to the Cu target. Argon gas is directed to the surface of each target.

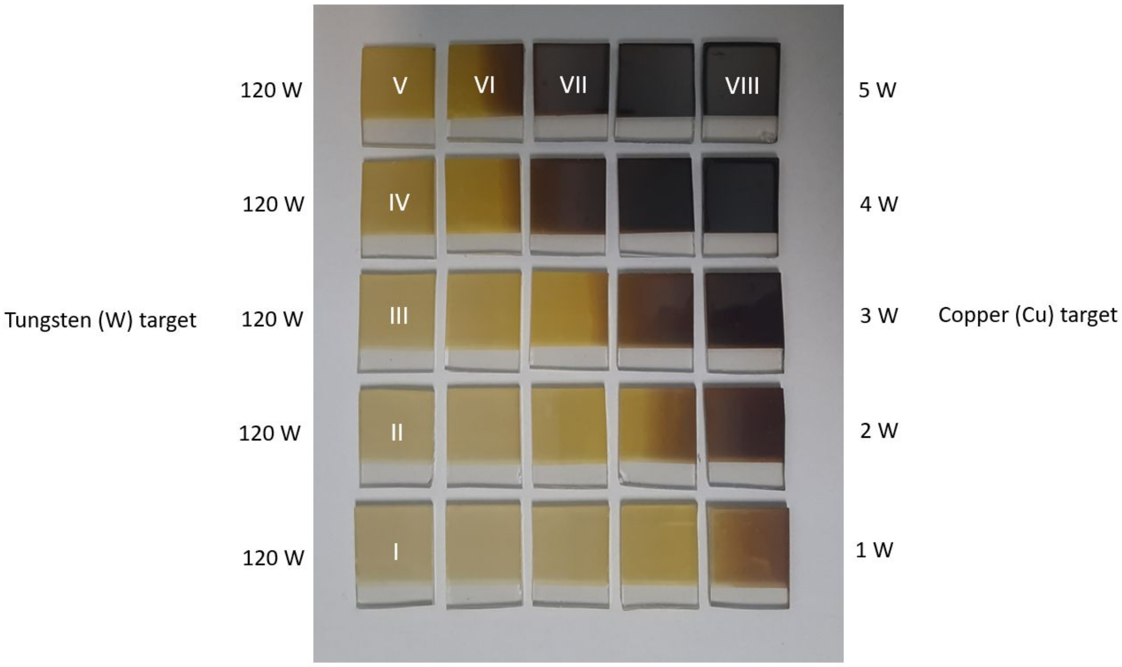

Figure S2. Set of 25 samples deposited on FTO substrate divided into 5 depositions. The power is fixed at the tungsten target (120W) and a variable power (from 1 W to 5 W) at the copper target. Each line shows one single deposition.

Table S1. Chemical composition of samples (atom%) from EDS.

	Atomic quantity (%)		
	Cu	W	O
Sample 1	3.22	32.8	63.98
Sample 2	3.76	31.83	64.41
Sample 3	5.17	29.84	65.00
Sample 4	8.27	28.81	62.92
Sample 5	13.27	25.50	61.23
Sample 6	20.92	20.83	58.25
Sample 7	26.27	16.67	57.06
Sample 8	41.15	6.86	52.00

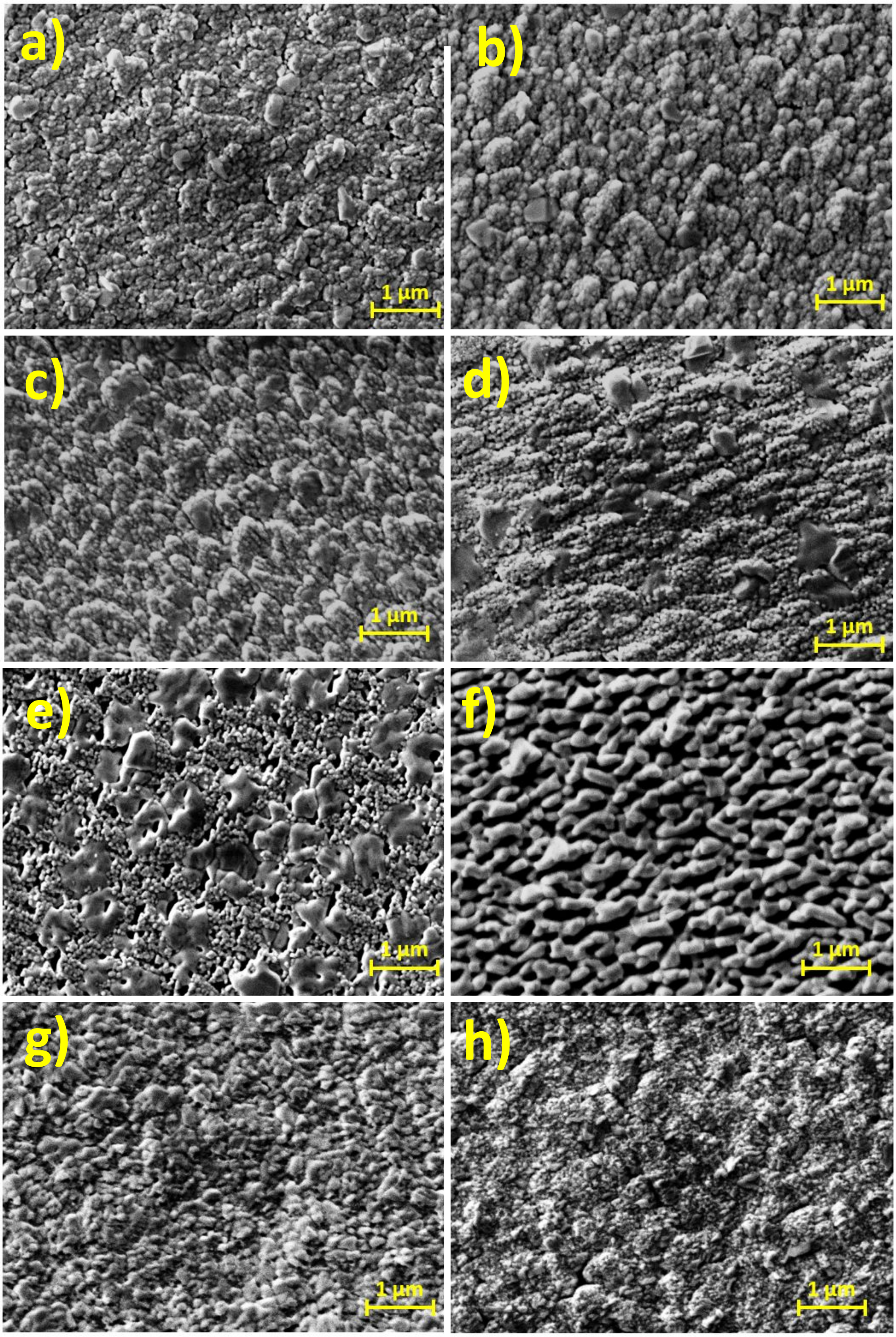

Figure S3. SEM images for the annealed samples on the FTO substrate. a) Sample I, b) Sample II, c) Sample III, d) Sample IV, e) Sample V, f) Sample VI, g) Sample VII, and h) Sample VIII. **Figure S3a**, **S3b,** and **S3c** show the films with preferred direction orientation with 200-400 nanometer grains. **Figure S3d** and **S3e** show 50 nanometers little grains with some plateaus. Those plateaus are probably linked to the $CuWO_4$ formation. **Figure S3f** displays homogeneous 400-500 nanometer grains with some degree of

porosity. And **Figure S3g** and **S3h** appear to have a smooth surface with compact 50-100 nm grains. Overall, all of them are compact and have some similarity in the morphology and grain size.

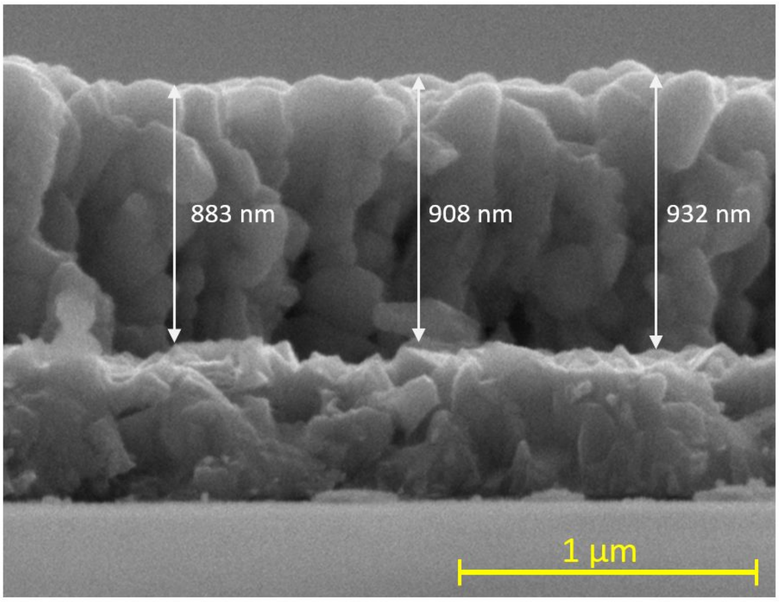

Figure S4.Cross-section SEM image from sample V on FTO substrate.

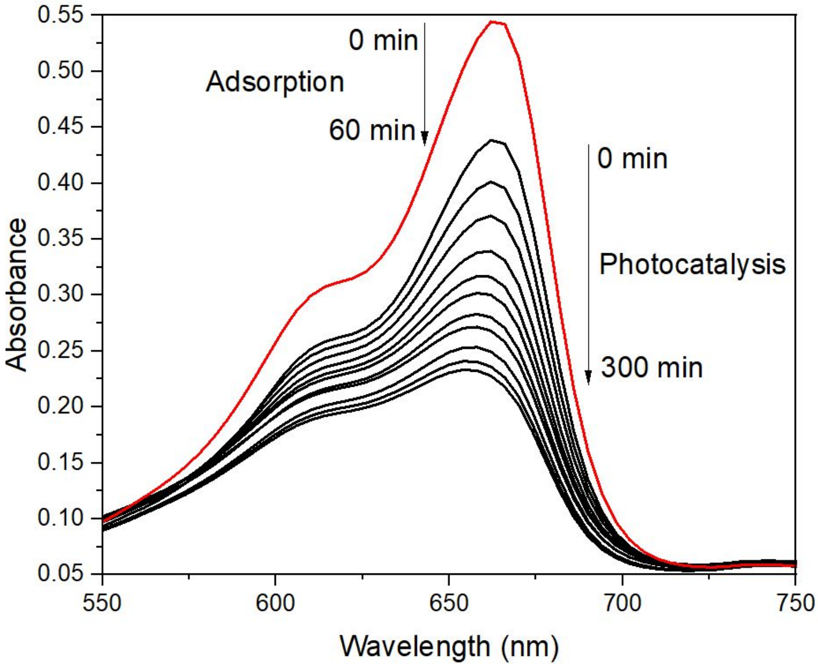

Figure S5. UV–Vis absorption spectra of methylene blue (MB) solution during adsorption and photocatalytic degradation experiments obtained for sample V deposited on SiO_2 substrate. The initial adsorption stage (0–60 min, dark conditions) shows a decrease in the chromophoric absorption band, indicating dye adsorption onto the catalyst surface. Subsequent irradiation (0–300 min) leads to a progressive reduction of the main absorption peak, confirming photocatalytic degradation. The red curve represents the initial MB solution prior to adsorption.

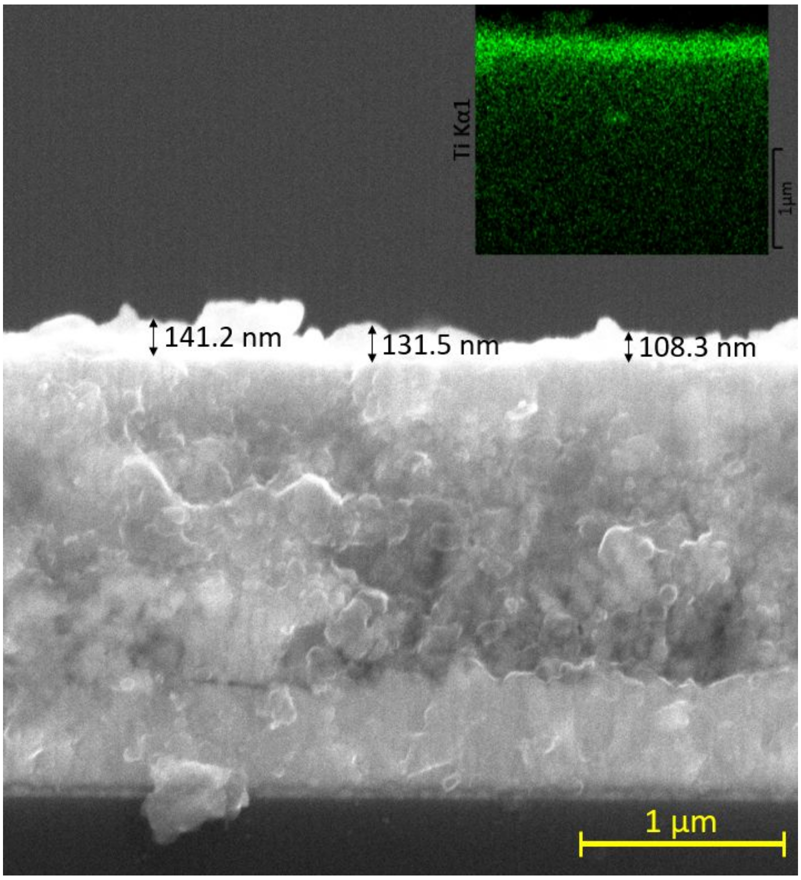

Figure S6. Cross-section SEM image from sample $WO_3/CuWO_4/TiO_2$ – 128 nm. The brightest part of the image represents the TiO_2 overlayer.

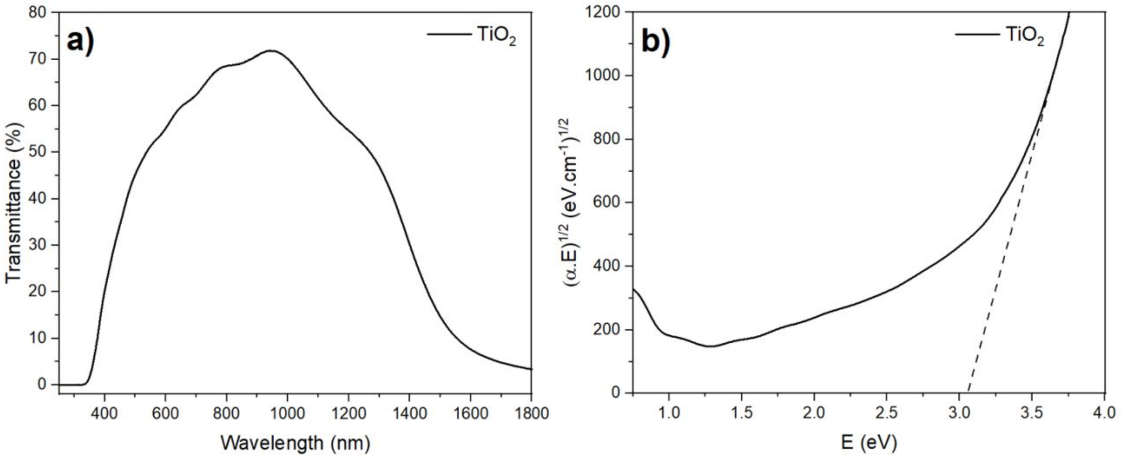

Figure S7. Transmittance data from the TiO_2 sample in a) and b) Tauc's plot showing a 3.05 eV TiO_2 bandgap.

Table S2. Stability and recyclability results for the samples IV and V deposited on FTO and SiO_2 substrates after three photocatalytic cycles.

	Cycle 1 (%)	Cycle 2 (%)	Cycle 3 (%)
**Sample IV on FTO**	42.5	40.4	41.9

**Sample V on FTO**	47.6	48.9	47.9
**Sample IV on SiO_2**	48.0	46.8	47.6
**Sample V on SiO_2**	50.3	50.4	50.7

Table S3. RMS values from AFM measurements

Samples	RMS (nm)
TiO_2	20.07
$WO_3/CuWO_4$	29.81
$WO_3/CuWO_4/TiO_2$ – 2 nm	28.11
$WO_3/CuWO_4/TiO_2$ – 4 nm	30.64
$WO_3/CuWO_4/TiO_2$ – 8 nm	27.31
$WO_3/CuWO_4/TiO_2$ – 16 nm	28.04
$WO_3/CuWO_4/TiO_2$ – 48 nm	29.32
$WO_3/CuWO_4/TiO_2$ – 64 nm	29.56
$WO_3/CuWO_4/TiO_2$ – 96 nm	28.24
$WO_3/CuWO_4/TiO_2$ – 128 nm	28.69

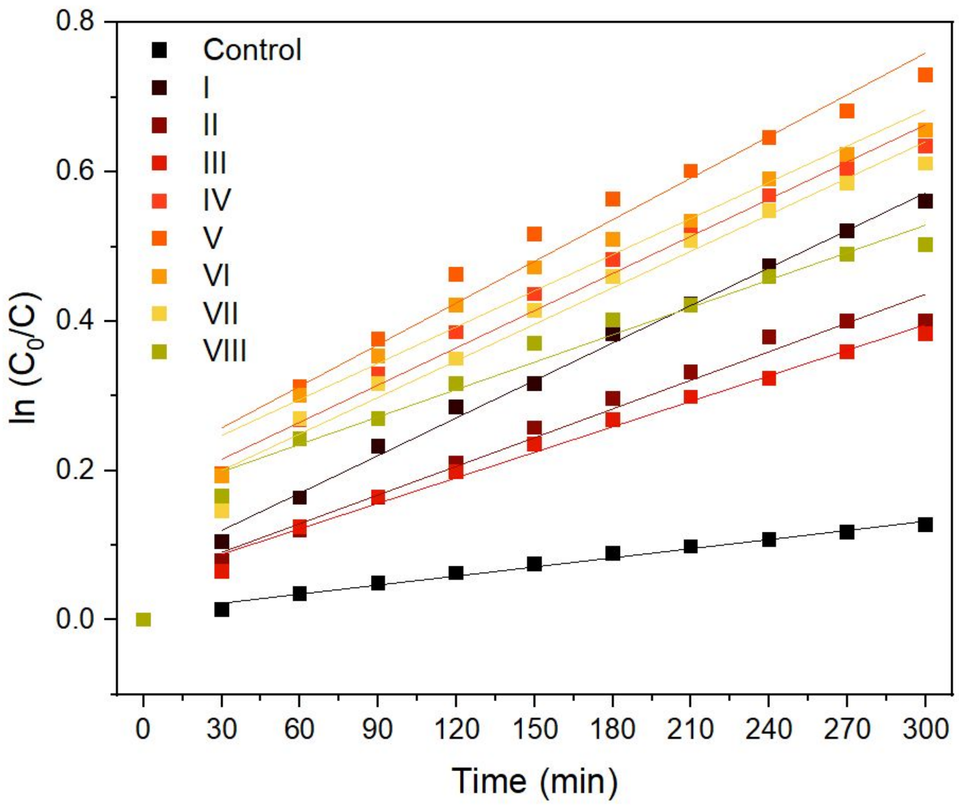

Figure S8. Pseudo-first-order (PFO) kinetic plots for photocatalytic degradation, represented as $\ln(C_0/C)$ versus irradiation time for the control sample and samples I–VIII on FTO substrate.

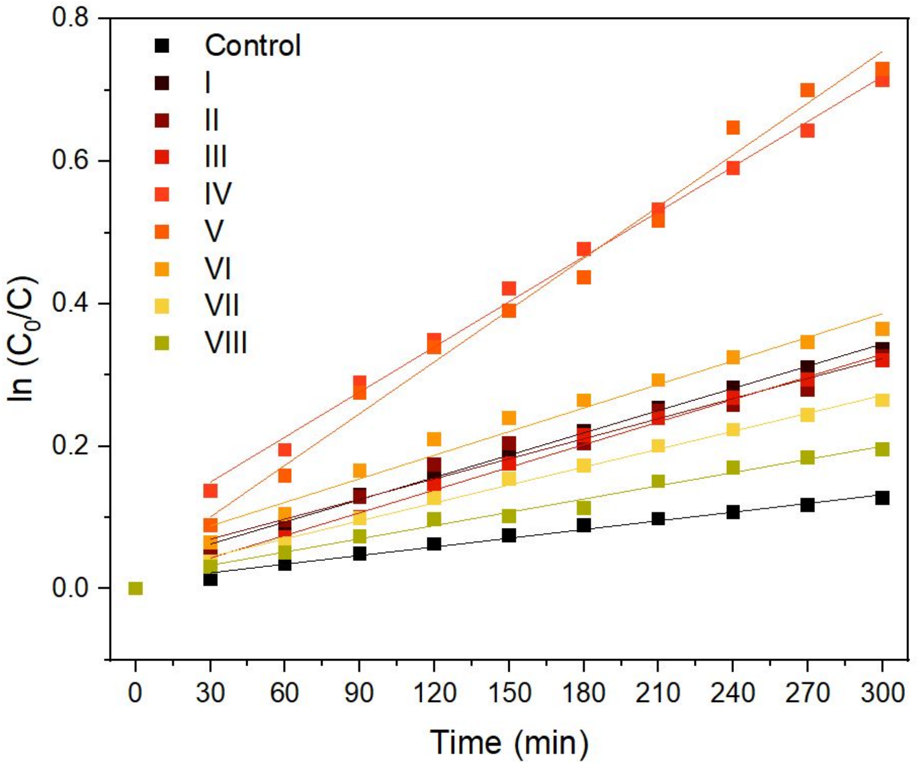

Figure S9. Pseudo-first-order (PFO) kinetic plots for photocatalytic degradation, represented as $ln(C_0/C)$ versus irradiation time for the control sample and samples I–VIII on SiO_2 substrate.

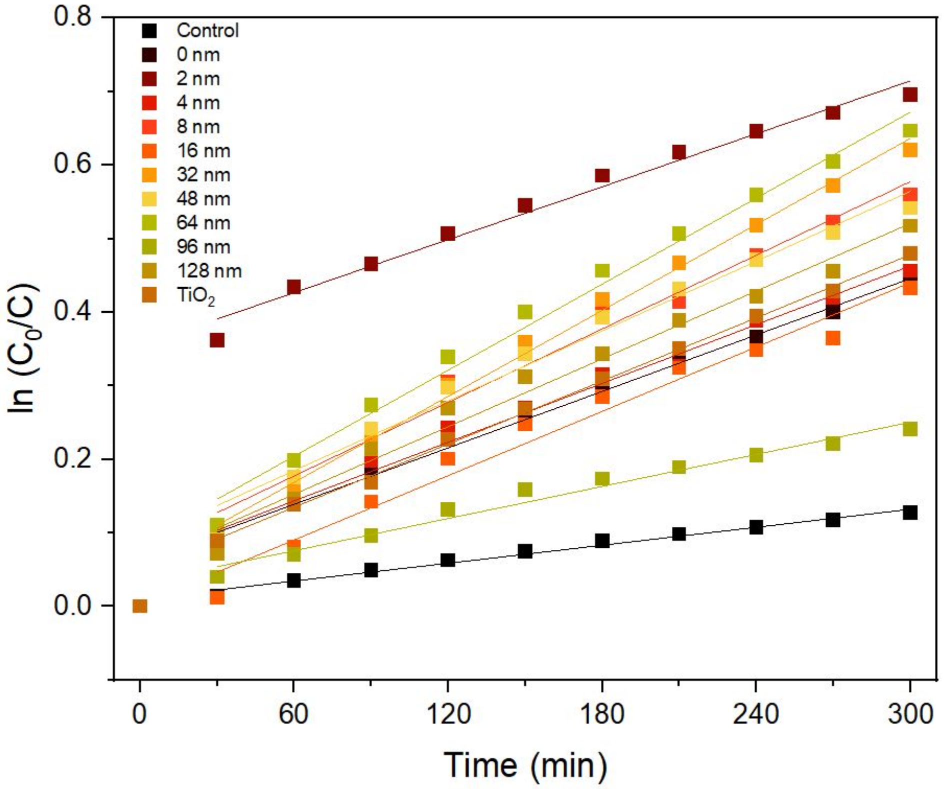

Figure S10. Pseudo-first-order (PFO) kinetic plots for photocatalytic degradation, represented as $ln(C_0/C)$ versus irradiation time for the control sample, $WO_3/CuWO_4$ sample, and $WO_3/CuWO_4/TiO_2$ samples.
